# Congenital malaria and neonatal bacterial co-infection in twins prematurely born to a mother with sickle-cell anaemia in the Democratic Republic of the Congo

**Published:** 2017-08-01

**Authors:** Junior E. Mudji, Johannes Blum, Timothy D. Rice, Frederick N. Baliraine

**Affiliations:** 1 Hôpital Evangélique de Vanga, Vanga, Democratic Republic of the Congo; 2 Swiss Tropical and Public Health Institute, Basel, Switzerland; 3 The University of Basel, Basel, Switzerland; 4 Saint Louis University School of Medicine, St. Louis, Missouri, USA; 5 LeTourneau University, Longview, Texas, USA

## Abstract

**Background:**

We report cases of gestational and congenital malaria with twin prematurity, low birth weight and bacterial co-infection. Congenital malaria is often misdiagnosed for lack of specific symptoms and a general lack of awareness of this presumably uncommon condition, and its diagnosis and prognosis become even more complex in the event of bacterial co-infections.

**Case presentation:**

A 35-weeks pregnant woman with sickle-cell disease and a history of spontaneous abortions was admitted at Vanga Hospital in DR Congo. She had fever (38.9°C) and microscopy-confirmed *P. falciparum* malaria and was put on 80/480 mg artemether-lumefantrine. She soon went into active labour, during which both twins developed acute foetal distress and were promptly delivered by C-section. The twins were underweight, and both had *P. falciparum* malaria at birth and were given 20 mg quinine twice daily. Both developed fever on the third day; a bacterial infection was suspected and 200 mg ceftriaxone was added to their treatment. Fever in both twins quickly resolved, and one twin totally recovered within 2 days of antibiotic treatment. The other twin developed acute respiratory distress and hypoxia and died.

**Discussion:**

This is a case of gestational and congenital malaria with prematurity, low birth weight and bacterial co-infection, but the patients were initially only treated for malaria based on their malaria-positive blood smears at birth.

**Conclusions:**

In malaria-endemic areas, babies should be screened for congenital malaria. Even with a confirmed malaria infection in the new-born, it is important consider the possibility of bacterial co-infections.

## 1 Introduction

Malaria endangers 125 million pregnancies annually, often causing adverse outcomes for both mother and foetus, including anaemia, intrauterine growth retardation, prematurity, low birth weight (LBW), and death. In sub-Saharan Africa, congenital malaria kills up to 200 thousand new-borns annually [1]. Malaria is deemed congenital if it is diagnosed in the new-born within seven days of birth [2]. Congenital malaria is the most neglected and least known aspect of malaria in terms of clinical manifestation and treatment, due to the long-held belief that it is extremely rare, particularly in endemic areas due to the protective effect of maternal immunity after birth [2,3]. However, incidences of 7-10% have been observed in parts of Africa [3].

## 2 Case presentation

On 20 November 2016, a febrile, 25-year-old, 35-week pregnant woman was admitted for the fourth time during the pregnancy at Vanga Hospital, Vanga, DR Congo. She had sickle-cell disease, with a history of several crises over the years and two previous spontaneous abortions (in 2012 and 2014). She obtained a bednet at her first antenatal visit, and was followed thereafter. She took 5 mg folic acid daily, and received prophylactic doses of sulfadoxine-pyrimethamine in weeks 16 and 26. Overall, she was hospitalised thrice for malaria during the 1^st^, 2^nd^ and 3^rd^ trimester, and once during the 2^nd^ trimester for a sickle-cell crisis (without malaria) and was treated with blood transfusion. The first malaria hospitalisation was at 12 weeks, followed by a sickle-cell crisis at 19 weeks, then malaria again at weeks 21 and 35. She was treated with 500 mg quinine twice daily for 7 days during the first two malaria episodes. Her thick and thin blood smears were malaria-negative after each course of treatment. She reported to use her bednet regularly, and our nurse verified the presence of the net at her home during the second hospitalisation. The physical exam upon her final admission revealed fever (38.9°C), an elevated respiratory rate (27 cycles/minute), and marginal hypotension (100/60 mmHg). Her palpebral and bulbar conjunctivae were pale and anicteric, respectively. The fundal height was 36 cm, foetal echocardiograms were normal, and no uterine contractions were detected. Her blood smears were positive for *P. falciparum*. The patient was stable overall. We administered 80/480 mg artemether-lumefantrine (Coartem®) for 3 days with 1000 mg paracetamol start and as needed for the fever. On the 5^th^ day of admission, she went into active labour. During labour, both twins developed acute foetal distress, indicated by tachycardia. A C-section was promptly performed to save the babies. Their new-born exam results are in Table 1.

**Table 1:** Physical examination results for prematurely born twins to a mother with sickle-cell disease in DRC.

Parameter	Twin-A	Twin-B	Normal Range [12,13]*	Comments
Gestational age at delivery (weeks)	35.7	35.7	≥37	Prematurity
Gender	Female	Female	Male or Female	Normal
Birth weight (kg)	1.8	2.2	2.5-5.0	Low birth weight
Apgar score (min. 1)	6	7	7-10	Twin-A: low,
				Twin-B: normal
Apgar score (min. 5)	8	9	7-10	Normal
Head circumference,	34 (50)	34.5 (69.8)	3-97	Normal
cm (percentile)				
Body length, cm (percentile)	46 (4.6)	46 (4.6)	3-97	Normal
Neurologic exam	Abnormal due to hypotonia	Abnormal due to hypotonia	Normal	Abnormal
Abdominal exam	Hepatosplenomegaly	Hepatosplenomegaly	Normal	Abnormal
Heart exam	Normal	Normal	Normal	Normal
Heart rate, beats/min.	142	150	110-180	Normal
Lung exam	Normal	Normal	Normal	Normal
Respiratory rate, breaths/min.	46	40	25-68	Normal
Body temperature, °C (day 0)	36.0	36.0	36.1-37.9	Normal
Body temperature, °C (day 3)	39.2	39.0	36.1-37.9	Fever
O_2_ saturation level, %	96	96	95-100	Adequate
Congenital malformations	None	None	None	Normal
Thin and thick blood smear (day 0)	*P. falciparum*	*P. falciparum*	No infection	Congenital malaria
Thin and thick blood smear (day 3)	Negative	Negative	Negative	Treatment effective
Blood group	B +	B +	None	Normal
Haemoglobin level, g/dl	19.8	18.3	14-24	Normal
Leucocyte count/mm^3^	13,700	12,700	9000-30,000	Normal day 0 values
Leucocyte formula, %	N 65, L 23, E 5	N 60, L 39, E 1	N 38-68, L 26-36, E 0-3	Eosinophilia in twin-A, lymphocytosis in twin-B
Casual blood glucose, mg/dl	89	70	30 -150	Normal

*Sources of growth chart and normal paediatric complete blood count data.

The mother was malaria-negative 7 days after treatment, but her twins had malaria at birth. They received 20 mg quinine intravenously twice daily, and 2 mg vitamin K intramuscularly for 2 days. However, on their third day of life, both developed fever but their blood smears were malaria-negative. A bacterial infection was suspected, as prematurity and LBW are commonly associated with neonatal infection [4,5]. Both twins were prescribed a daily, 7-day long intravenous course of 200 mg ceftriaxone, besides quinine.

Fever in both twins quickly resolved, and twin-A totally recovered within 2 days of antibiotic treatment. Twin-B, however, who also had lymphocytosis, developed acute respiratory distress and hypoxia. Her oxygen saturation rapidly dropped from >90% to 60% in 2 hrs. Oxygen was given at 1 l/pm by nasal cannula to keep the saturation above 90%. The next day, she died of respiratory distress.

## 3 Discussion

Clearly, this is case of gestational and congenital malaria with prematurity, LBW (normal range 2.5-5 kg, Table 1) and possible bacterial co-infection. LBW is a common finding in premature babies of mothers with sickle-cell disease and gestational malaria. Factors such as prematurity, acute anaemic episodes and the number of sickle-cell-related complications during pregnancy may be responsible for the LBW [6].

It was easy to determine that the twins had congenital malaria, since their blood smears were done immediately after birth and both were positive for *P. falciparum,* the same parasite species identified in their mother. However, congenital malaria is often misdiagnosed for lack of specific symptoms and a general lack of awareness of this presumably uncommon condition [2]. The most common clinical features are fever, anaemia, and splenomegaly. Whereas these signs can be observed in a day-old baby, they may take weeks or even months to manifest [7]. Other signs include hepatomegaly, jaundice, regurgitation, loose stools, poor feeding, drowsiness, restlessness, and cyanosis [2,7]. The delay in disease onset has been attributed to immunological factors that may initially protect babies, particularly those born to mothers residing in malaria-endemic areas [2]. Our twins initially only presented with splenohepatomegaly, without fever (Table 1). We administered antimalarial treatment solely based on their malaria-positive blood smears.

Cases of malaria-bacteria co-infection have been reported elsewhere in Africa [2,8], but we did not test for bacteria due to our resource-limited capacity to do microbiological cultures at our hospital. Nevertheless, we believe the fever in both twins was caused by a bacterial infection. This is because the fever came a few days after the beginning of malaria treatment, their blood smears were by then malaria-negative, and the fever in both quickly resolved upon beginning antibiotic treatment. Moreover, whereas twin-B developed and succumbed to acute respiratory distress, twin-A (who had no respiratory distress), fully recovered soon after starting antibiotic treatment. In fact, congenital bacterial infections are more common in preterm babies than previously thought [9], and sepsis is the most common cause of acute respiratory distress syndrome [10]. The source of infection was probably the same for both twins as they became febrile at the same time. We did not begin antibiotic treatment at birth because we considered malaria as the sole cause of the prematurity, based on their malaria-positive blood smears.

Notably, the mother contracted malaria despite having a bednet and receiving intermittent preventive treatment (IPTp) with sulfadoxine-pyrimethamine. Whereas the second malaria crisis may be attributed to the blood transfusion since malaria was diagnosed two weeks thereafter, her 1^st^ and 3^rd^ malaria crises involved no transfusion. In all cases, the drugs were deemed effective, since all blood smears were malaria-negative at the end of each treatment. The current DRC national guidelines for prevention of malaria in pregnancy are focused on IPTp and bednets, but this strategy may not always be effective. Although we verified the presence of a bednet in the patient’s house, we cannot guarantee that she used it regularly. In multiple African settings bednet usage among pregnant women, for a variety of reasons including discomfort, runs below 60% [11].

## 4 Conclusions

Babies in malaria-endemic areas, particularly those of mothers with a history of gestational malaria, should be screened for congenital malaria. In cases of prematurity, even with a confirmed malaria infection in the new-born, it is important to consider the possibility of bacterial co-infections. When in doubt, antibiotics should also be administered at the doctor’s discretion, particularly in healthcare facilities with limited laboratory capacity to diagnose bacterial infections. Moreover, there is need to investigate the possibility of drug resistance to sulfadoxine-pyrimethamine in the area, as it did not protect the mother from malaria.
